# Revaccinations in the Danish Army in 1837-8

**Published:** 1840-01

**Authors:** 


REVACCINATIONS IN THE DANISH ARMY IN 1837-38.

| Year. | No. Revaccinated. | Cicatrices of former Vaccination. | | Result of Revaccination. | | |
|---|---|---|---|---|---|---|
| | | Distinct. | Not Distinct. | Genuine Pustules. | Not Genuine Pustules. | No Result. |
| 1837 | 3741 | ... | ... | 1601 | 1015 | 1125 |
| 1838 | 3721 | 3317 | 404 | 1464 | 1000 | 1257 |

Of the number who in 1838 resisted revaccination, 129 had had the natural smallpox, and 111 had been twice revaccinated. In the year 1838, there were, in the whole army, twenty-four cases of smallpox, partly children. Of the individuals who have been successfully revaccinated, not one has been attacked with smallpox."—*Bibliothek for Læger*, 1839.

